# Porcine Hokovirus in Domestic Pigs, Cameroon

**DOI:** 10.3201/eid1912.130891

**Published:** 2013-12

**Authors:** Cornelia Adlhoch, Marco Kaiser, Manchang T. Kingsley, Norbert Georg Schwarz, Markus Ulrich, Vanessa S. de Paula, Julian Ehlers, Anna Löwa, Achukwi M. Daniel, Sven Poppert, Jonas Schmidt-Chanasit, Heinz Ellerbrok

**Affiliations:** Robert Koch Institute, Berlin, Germany (C. Adlhoch, M. Ulrich, A. Löwa, H. Ellerbrok);; GenExpress, Berlin (M. Kaiser);; Bernhard Nocht Institute for Tropical Medicine, Hamburg, Germany (N.G. Schwarz, J. Ehlers, S. Poppert, J. Schmidt-Chanasit);; Institute of Agricultural Research for Development, Ngaoundere, Cameroon (M.T. Kingsley, A.M. Daniel);; Fundação Oswaldo Cruz, Rio de Janeiro, Brazil (V.S. de Paula)

**Keywords:** pigs, PHoV, Cameroon, porcine hokovirus, partetravirus, PARV4, viruses

**To the Editor:** Since 2005, new parvoviruses forming a novel genus of the proposed name *Partetravirus*, within the subfamily *Parvovirinae*, have been described (*1*). Human parvovirus 4 (PARV4) with 3 different genotypes globally infects humans (*2*). A related porcine virus, hokovirus (HoV or porcine partetravirus), was found in wild boar and domestic pig populations in Germany, Romania, China, and the United States, with prevalences of 12%–47%, forming 1 common genotype (*3*–*6*). Prevalence figures from sub-Saharan Africa are not available. Furthermore, no information about possibly region-associated genotypes is available for porcine HoV, although it is for human PARV4 from the same genus. We therefore used samples (collected during February–March 2012) from a study investigating hepatitis E virus (HEV) in pigs from Cameroon (*7*) to analyze the occurrence of porcine HoV in pigs in Africa and to determine the respective genotype.

Viral DNA was extracted from liver samples by using the RTP DNA/RNA Virus Mini Kit II (STRATECMolecular, Berlin, Germany) according to the manufacturer’s instructions. DNA samples were pooled, with each pool containing 3 different samples. A total of 94 pooled samples from 282 animals originating from 3 districts in Cameroon (Doula, Yaoundé, and Bamenda) were investigated by using quantitative real-time PCR (*3*,*7*). Samples from pools that tested positive were analyzed individually.

We detected HoV in 65 (69%) of the 94 pooled samples: 2 (15%) of 13 from Bamenda, 39 (70%) of 56 from Douala, and 24 (96%) of 25 from Yaoundé. We used an online tool to estimate the individual prevalence from pooled samples for fixed pool size and perfect test with exact 5% upper and lower CIs (http://epitools.ausvet.com.au/content.php?page = PooledPrevalence). A pool size of 3 with a total of 94 pooled samples and 65 positive samples resulted in an estimated general prevalence of 32.4% (95% CI 27%–39%). For Bamenda, the estimated prevalence was 5.4% (95% CI 1%–16%); for Douala, 32.8% (95% CI 25%–41%); and for Yaoundé, 65.8% (95% CI 44%–87%).

From 94 positive pools, a total of 184 samples were available for individual testing: 6 from Bamenda, 110 from Douala, and 68 from Yaoundé; 12 were missing. Using the results from the negative tested pools and the individual testing, we found an estimated general prevalence of 47% (128/270). The regional prevalence was 10% (4/39) for Bamenda, 41% (65/160) for Douala, and 83% (59/71) for Yaoundé.

These prevalences are higher than the estimates, but lie within the regional estimates within the range of the CI determined with the online tool. The discrepancy in the total prevalence might be due to the missing samples for the individual testing. Our results show that pooled sample testing can yield a good approximation of the actual prevalence, at least for settings in Africa. The varying prevalence and inhomogeneous regional distribution of porcine HoV correspond to previous findings from Europe, China, and the United States in wild boar and domestic pigs (*3*,*5*,*6*). Overall, no general defined pig-breeding program is in place in Cameroon. Douala and Yaoundé are the main markets for pig trade. Yaoundé, the main town for pig purchase and slaughter, gets live pigs from northwestern (Bamenda), western, and northern Cameroon, and Douala receives pigs from northwestern (Bamenda), western, and southwestern Cameroon. To fully understand the observed regional prevalences, the presence of HoV needs to be investigated in detail in the southwest, west, and north, where intensive farming systems are in place and pig farming is of economic importance.

Near full-length genome data were generated from 3 positive samples, and partial sequence information was retrieved for 8 additional samples (Figure) as described (*3*). The phylogenetic analysis showed a very close relation, with 98%–99% homology between the porcine HoV isolates from Cameroon, Europe, the United States, and China. In contrast to bovine HoV or PARV4 in humans and chimpanzees, only 1 porcine genotype has been observed worldwide, which might point to 1 common ancestor (*8*,*9*). Because the liver samples were taken from European landrace or cross-bred pigs, the common ancestor of HoV might have originated from Europe. Pigs chronically infected with HoV could have been imported from Europe to Cameroon during the extensive global industrial farming and pig trade in the last century. The detection of the European HEV genotype (gt) 3 instead of the “traditional” African gt1 and gt2 in these pigs supports this hypothesis (*7*,*10*). Nevertheless, the high grade of homology of porcine HoV found worldwide suggests a highly species-specific virus with a low mutation frequency. To study the circulating genotypes in Africa and to generate hypotheses on the influence of trade, researchers need to college data from other African countries. However, data on HoV in wild animals, such as warthogs and pot-bellied pigs, are limited and need to be addressed to elucidate the genomic intravariability and intervariability of this new family of parvoviruses.

**Figure:**
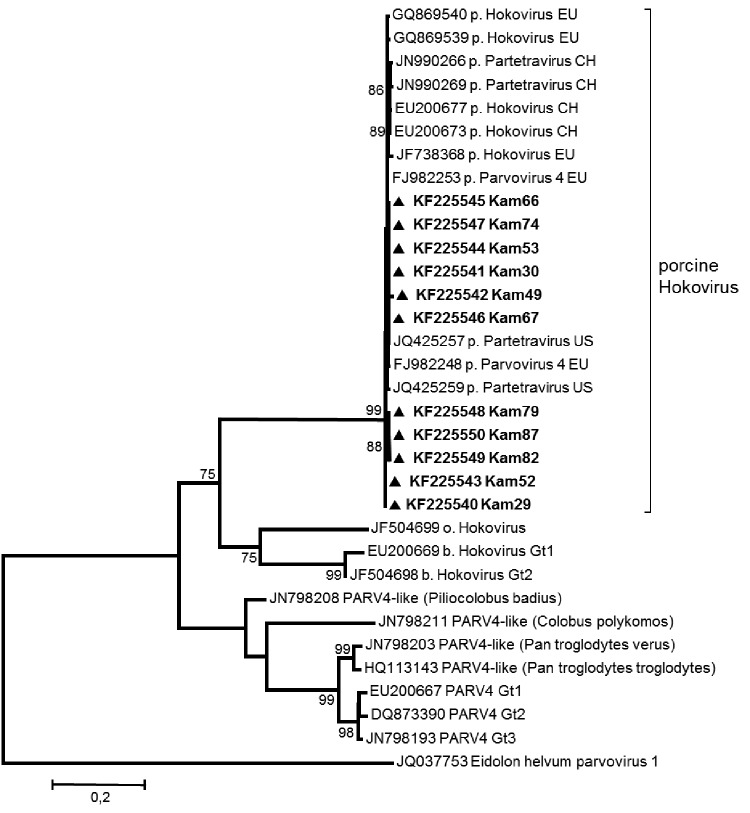
Phylogenetic tree of near full-length (4.7 kbp) and partial sequences (open reading frame 2, 0.4–1.9 kbp) of porcine hokovirus (HoV)/partetraviruses (PtV) created by using MEGA5.1 (www.megasoftware.net) with the maximum-likelihood method (GTR+G+I) and bootstrap analysis of 500 resamplings. New sequences from Cameroon are shown in **boldface**. EU, Europe; CH, China; US, United States; Gt, genotype; PARV4, parvovirus 4. Scale bar indicates nucleotide substitutions per site.
